# Editorial: Molecular mechanisms of cilia related diseases

**DOI:** 10.3389/fmolb.2024.1421419

**Published:** 2024-05-23

**Authors:** Sara Carvalhal, Bruno Carmona, Anne-Marie Tassin, João Gonçalves

**Affiliations:** ^1^ Algarve Biomedical Center Research Institute (ABC-Ri), Universidade do Algarve, Faro, Portugal; ^2^ Algarve Biomedical Center (ABC), Universidade do Algarve, Faro, Portugal; ^3^ Escola Superior de Tecnologia da Saúde de Lisboa, Instituto Politécnico de Lisboa, Lisboa, Portugal; ^4^ Centro de Química Estrutural, Institute of Molecular Sciences, Faculdade de Ciências da Universidade de Lisboa, Lisboa, Portugal; ^5^ Institute for Integrative Biology of the Cell (I2BC), Commissariat à l’énergie atomique (CEA), Centre national de la recherche scientifique (CNRS), Université Paris Sud, Université Paris-Saclay, Gif-sur-Yvette, France; ^6^ Evotec, Campus Curie, Toulouse, France

**Keywords:** cilium, ciliopathies, molecular mechanisms of disease, rare genetic diseases, organelle; cytoskeleton; signalling

Eukaryotic cilia are fascinating evolutionarily conserved microtubule-based organelles that protrude from the cell surface. In vertebrates, multiple types of motile and primary (immotile) cilia fulfill motility and signaling functions, critical for embryonic development and homeostasis of adult tissues. Importantly, perturbed cilia assembly and functions are associated with a growing number of diseases. This Research Topic gathers an update on recent progress made in understanding the molecular mechanisms of cilia-related diseases. Critically, understanding disease development has been facilitated by advances in technology. For example, the importance of omics techniques for monitoring the progression of cilia-associated rare diseases is showcased in the work of (Jeziorny et al.). This study applied an untargeted metabolomic approach using LC-QTOF-MS to study patients with Alström (ALMS) and Bardet-Biedl (BBS), which shared defective primary ciliary structures and found common molecular fingerprints between ALMS and BBS, and alterations in various lipid metabolites when comparing obese and healthy participants (Jeziorny et al.).

Primary cilia are essential sensory and signaling organelles, whose dysregulation is implicated in well-established ciliopathies, rare genetic multisystemic human disorders, as well as in more common and broader diseases like cancer (Lee), brain (Thirugnanam et al.; Yeo et al.), or cardiac diseases (Chen et al.). The role of cilia as signaling hubs is highlighted in the review by Flax et al., which focuses on understudied ciliary kinases and their involvement in neurological disorders, cancer development and ciliopathies (Flax et al.). The review presented by Lee synthesizes the current broad research elucidating the relevance of primary cilia-associated signaling pathways (e.g., Sonic hedgehog and Wnt) in the acquisition of drug resistance and explores potential future directions in overcoming it (Lee). Alzheimer’s disease (AD) is characterized by cognitive decline due to neuronal degeneration and amyloid plaque accumulation. Microglia, the brain’s immune cells, play a crucial role in clearing toxic proteins associated with AD. In their paper Yeo et al., report that primary cilia modulate microglial secretory function. Their transient presence in microglia, diminished in AD, influences extracellular proteostasis and neuroinflammatory responses, exacerbating the disease (Yeo et al.). Additionally, primary cilia in brain microvascular endothelial cells play vital roles in sensory perception, cell signaling, and vascular stability, as shown in (Thirugnanam et al.). Cilia emergence from both mother and daughter centrioles during the cell cycle, a rare observation, contributes to the 2-cilia phenotype observed in these cells during the G0/G1 phase. Disruption in proteins required for ciliogenesis affects ciliary dynamics and cellular function, potentially impacting vascular homeostasis. Moreover, the work by Chen and colleagues showed that nucleoporins (NUPs), exhibit non-classical functions at the ciliary base, influencing ciliary integrity and function, thus being implicated in congenital heart diseases and ciliopathies (Chen et al.). Understanding these non-classical roles of NUPs enhances comprehension of ciliopathy etiology and may offer insights into therapeutic interventions.

The identification of genetic variants is key to bring forth novel diagnostic markers and therapeutic targets and to elucidate cilia biology, the function of disease-associated genes, and the molecular mechanisms of cilia-associated diseases. In this Research Topic, a subset of articles discusses rare disorders, and describe the significance of genetic variants as novel biomarkers for their diagnosis (Walczak-Sztulpa et al.; Yuan et al.) and progression (Jeziorny et al.). Furthermore, Yin et al. and Schramm et al. explore symptom management in rare diseases. Specifically, these publications address novel findings in ALMS and BBS syndromes, Cranioectodermal Dysplasia (CED), left-right asymmetry disorders, and Primary Ciliary Dyskinesia (PCD). The work of Walczak-Szulpa et al. characterizes a new missense variant in WDR35 and showed that CED patient urine-derived renal epithelial cells carrying it presented ciliary phenotypes, reinforcing its involvement in pathogenesis and its relevance as a novel biomarker for chronic kidney disease (Walczak-Sztulpa et al.). Regarding left-right asymmetry disorders, Yuan et al., identified novel compound variants in DNAH1, a gene associated with PCD and other related disorders. These variants shed light on the significance of DNAH1 in the ciliary structure and its associated diseases’ clinical manifestations, particularly infertility (Yuan et al.). Still in the context of cilia in vertebrate embryo left-right asymmetry, Abdel-Razek et al. used zebrafish as a model to study the mechanisms underlying the formation of the ciliated “left-right organizer” (LRO). Importantly, they describe that Ca+ signaling mediated by the sarcoplasmic/endoplasmic reticulum Ca2+-ATPase (SERCA) regulates mitosis in the precursor cells of the ciliated LRO (Abdel-Razek et al.). PCD is strongly associated with Chronic Rhinosinusitis (CRS). Yin et al. and Schramm et al., employed non-invasive imaging techniques to examine the impact of CRS in mice with a deletion in the axonemal dynein Dnaic1 gene (Yin et al.), or imaging data from individuals with genetically confirmed PCD carrying pathogenic mutations in different genes (Schramm et al.). Although further research is needed, these studies underscore the importance of incorporating chronic respiratory symptoms into the diagnostic evaluation and disease management in PCD patients. Finally, highlighting the importance of understanding gene function and the impact of pathogenic mutations on disease development, we have the studies of (Garfa Traoré et al. and Hoffman et al.). The ciliary transition zone (TZ) is a ciliopathy hotspot, with most of its resident proteins being coded by ciliopathy genes. Garfa Traoré et al. showed that Nephronophthisis-associated TZ proteins NPHP1 and NPHP4 play a role in cilia-mediated flow sensation in kidney cells, and that NPHP1-deficient cells are affected in ciliary length and actin regulation. The authors also identified the cholesterol biosynthesis and uptake pathway as a novel signaling pathway induced by shear stress (Garfa Traoré et al.). Lastly, the intraflagellar transport (IFT) machinery is crucial for cilia assembly and signaling protein trans-localization and involves proteins like TTC30A and TTC30B. Mutations in TTC30A affect ciliary localization of Smoothened, a key player in the Sonic hedgehog (Shh) pathway. In their article, Hoffman et al. highlight the intricate interplay between ciliary dynamics and developmental signaling. Impaired ciliary signaling pathways, such as Shh, are implicated in developmental disorders like synpolydactyly, a rare limb deformity (Hoffmann et al.).

In conclusion, understanding the roles of the multiple human cilia types in cellular function and disease pathogenesis opens new avenues for therapeutic interventions across various fields, from cancer treatment to neurodegenerative disorders and developmental anomalies. Continued research into ciliary biogenesis, maintenance and functions promises innovative strategies for addressing complex diseases and improving patient treatment outcomes.

